# A meta-analysis on the effectiveness of Traditional Chinese Medicine Emotional Nursing in improving anxiety and depression symptoms in elderly patients

**DOI:** 10.3389/fpsyg.2025.1535349

**Published:** 2025-06-19

**Authors:** Chunxiao Zhao, Xuelian Chen, Aihua Zhang, Ningya Huo, Siyao Yu, Lizu Lai, Jing Liu

**Affiliations:** ^1^School of Medical Humanities, Hubei University of Chinese Medicine, Wuhan, Hubei, China; ^2^Hubei Shizhen Laboratory, Wuhan, Hubei, China; ^3^Hubei Key Research Base of Humanities and Social Sciences, Wuhan, Hubei, China

**Keywords:** anxiety, depression, meta-analysis, TCM emotional nursing, the elderly

## Abstract

**Background:**

This study used meta-analysis to explore the effectiveness of Traditional Chinese Medicine (TCM) emotional nursing intervention on the anxiety and depression of the elderly and factors that might influence this intervention, so as to provide reference for optimizing the effect of TCM emotional nursing for the elderly patients.

**Methods:**

Randomized controlled trials of TCM emotional nursing on improving anxiety and depression symptoms in elderly patients were searched from PubMed, Web of Science, Cochrane Library, Science Direct, China National Knowledge Infrastructure (CNKI), Wanfang Standards Database, and CQVIP. The Cochrane Collaboration’ s tool was adopted for assessing risk of bias of studies, and CMA 3.3 was used for statistical analysis.

**Results:**

Thirty-nine literatures and 40 studies finally met the study standards, including 4,425 patients. The results showed that TCM emotional nursing could improve the anxiety and depression symptoms of the elderly patients with a significant large effect (anxiety: *g* = 2.14, depression: *g* = 2.18). Subgroup analysis and meta-regression analysis showed that scale type, sample size and age of subjects affected the effect of intervention on anxiety. Sample size, age of subjects and course of disease affect the effect of intervention on depression.

**Conclusion:**

Traditional Chinese Medicine emotional nursing can significantly improve the symptoms of anxiety and depression in the elderly. Future studies may further focus on the moderators of the Effectiveness of TCM emotional nursing intervention, and optimize the intervention plan.

## Introduction

China has been facing the challenge of an aging society since the beginning of the 21st century. The aging of the Chinese population continues to deepen, and it is about to step into a moderately aging society (Liu, 2021). With the more and more large aging population, the physical and mental health of the elderly has also attracted wider attention from the whole society. The prevalence of various types of physical diseases in the elderly continues to rise with age, which may not only bring physical pain to the elderly, but also cause mental obstacles such as anxiety, depression and so on (Cheng et al., 2023). Those mental disorders not only hinder the recovery of physical diseases, but also affect patients’ quality of life. Therefore, it is necessary to improve the mental health of the elderly patients as well as treating somatic diseases.

Medication alone can only improve the physical discomfort of the elderly, instead of the mental health of the elderly. It’s in need to supplement mental care to achieve the purpose of physical and mental treatment at the same time in medication. Currently, among all methods to improve patients’ mental state, Traditional Chinese Medicine (TCM) emotional nursing has gradually gained the attention of researchers and practitioners because of the effectiveness with its cultural acceptance and broad mass base. Different from conventional psychological care, based on the concepts of yin and yang balance, internal impairment caused by excess of seven emotions, and the generation and restriction of the five elements in Chinese medicine, TCM emotional nursing adopts methods of win-win situation of the affective and the emotional, empathy, and reasoning and enlightenment to improve patients’ mental state (Wang, 2015; Zhang and Jiang, 2021). In treatment of patients’ physical disease, the supplement of TCM emotional nursing helps patients improve their awareness of diseases, reduce their negative perspectives about the disease, which provides patients with mentally inner power when facing the challenge of diseases.

Some meta-analyses have explored the effect of TCM emotional nursing in the treatment of post-stroke depression and anxiety disorders, and the results showed that there was a significant difference in the improvement of patients ‘anxiety and depression between the TCM emotional nursing group and the control group (Li et al., 2020; Liang et al., 2015; Liu et al., 2021). Researchers also analyzed the intervention effects of TCM emotional nursing on mood disorders in cancer patients (Kang et al., 2017; Ma and Xiao, 2019) and chronic obstructive pulmonary disease patients (Kang et al., 2017), and found significantly effective intervention. However, previous meta-analyses did not differentiate intervention subjects, and thus those findings could not be applied to determine the valid intervention of TCM emotional nursing for aging patients. Moreover, there are lacks about factors that may affect the intervention such as the type of disease and the age of patients.

In summary, TCM emotional nursing intervention may be a promising therapy for mental health of the elderly patients, but there is still a lack of advanced evidence-based supports with high reliability. The aim of this study was to explore the effectiveness of TCM emotional nursing on elderly patients’ anxiety and depression symptoms via meta-analysis, and factors that may affect its validity, so as to provide some evidence-based supports to facilitate aging patients’ mental health.

## Method

This study was conducted and reported according to the criteria declared by Preferred Reporting Items for Systematic Reviews and Meta-Analyses (PRISMA) guidelines (Liberati et al., 2009) and preregistered with PROSPERO (CRD42023423865).

### Search strategy

Studies were searched in the following databases: PubMed, Web of Science, Cochrane Library, Science Direct China National Knowledge Infrastructure (CNKI), Wanfang Standards Database, and CQVIP. The search keywords used were: “traditional Chinese emotional nursing” OR “traditional Chinese emotional therapy” OR “emotions treatment of TCM (Traditional Chinese Medicine” OR “Chinese medicine psychological” OR “TCM psychotherapy” OR “TCM nursing” OR “TCM psychosomatic medicine” OR “TCM emotional therapy” OR “TCM emotional nursing” OR “TCM emotional therapy,” and “traditional Chinese medicine”). The search covered the literature up to July 2023. In addition, reference tracking was conducted by searching the reference lists of eligible studies and relevant review articles. Literature was screened by the first author, checked by the second author, and dissenting literature was resolved by consensus with the corresponding author.

### Inclusion and exclusion criteria

Literature inclusion and exclusion criteria for inclusion were: (1) elderly people suffering from physiological diseases. Definition of the elderly: individuals aged 60 years and above as defined in the “Law of the People’s Republic of China on the Protection of the Rights and Interests of the Elderly.” (2) intervention was TCM emotional nursing, which refers to a kind of Chinese medicine psychotherapy based on the principles of the Five Zang Organs, Five Emotions, and the inter-promoting and inter-restraining relationships of the Five Elements. The therapy aims to maintain patients’ positive attitudes toward treatment and long-term healthful emotions by improving their moods (Liu et al., 2023), with adjunct methods such as diet and physical exercise. (3) The control group included routine care group, waiting group, or active control group with other interventions. (4) The outcome variables were anxiety and depressive symptoms. There was no standardized requirement for the measurement instrument for the outcome variables, but they must be a scale with good reliability and validity. (5) The type of study was a randomized controlled trial (RCT) with both pre-test and post-test. (6) The included studies were peer-reviewed journals, excluding master’s and doctoral dissertations. (7) The language of the paper was either English or Chinese.

Exclusion criteria: (1) studies in which the intervention combined herbal medicine, acupuncture, or acupoint massage. (2) Studies targeting perioperative emotional interventions. (3) Pre-post trials, single-group pre-post tests, and RCTs with only post-test measurements were excluded.

**Figure 1:**
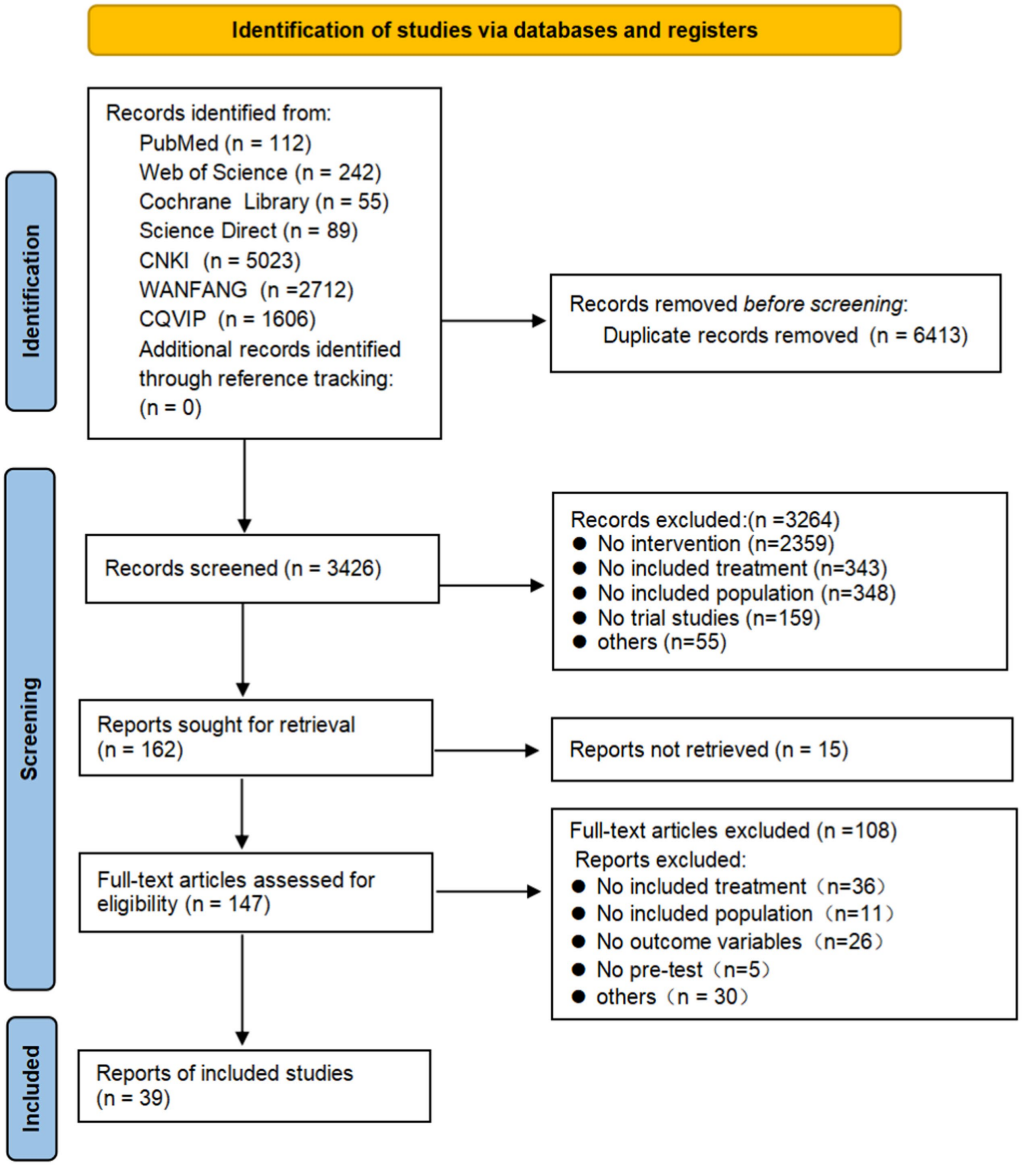
PRISMA flow diagram of the study.

The literature search and screening flowchart is shown in Figure 1.

### Meta-analysis procedure

Comprehensive Meta-Analysis Version 3.3 (CMA 3.3) was used for meta-analysis (Borenstein et al., 2021).

#### Data extraction and study quality assessment

Characteristics of each literature and outcome variables included in the analysis were extracted and coded. The process involved two authors independently extracting and coding the data, with final codes determined in consultation with the corresponding author for those with disagreements. Literature characteristics codes included: first author, year of publication, year of study, sample size, type of disease, age of subjects (years), duration of disease, outcome variable, type of control group, and measurement tool. Specific information is shown in Supplementary Table 1. Outcome data included: pre-test mean and standard deviation of the intervention group, post-test scores and standard deviation, and sample sizes of the intervention and control groups.

This study used the Cochrane Collaboration’s risk assessment tool (Higgins et al., 2011) to assess the risk of bias of the included studies. The risk of bias was assessed across the following six categories: selection bias (generation of randomized sequences, allocation concealment), performance bias, detection bias, attrition bias, reporting bias, and other potential sources of bias. Each category was assessed as low risk, high risk or unclear risk.

The details are as follows:

Selection bias consisted of two components: (1) random sequence generation: Did the researcher use a random sequence for group allocation? (2) Allocation concealment: Was the intervention allocation visible before or during the participants’ enrollment?

Performance bias: assess whether the implementer and participants were double-blinded to the implementation and group allocation in the experiment.

Detection bias: assesses whether the investigators who measured the results were aware of the intervention allocation.

Attrition bias: evaluate whether there were systematic between-group differences in the number of dropouts.

Reporting bias: assessing whether the results were selectively reported.

Other bias: bias not covered in the above that could affect the objectivity of the study results, such as between-group differences in subjects’ baseline characteristics despite randomized sequential grouping.

Data extraction and quality assessment were conducted by the first and second authors separately, and disagreements were resolved by consensus with the corresponding author.

#### Effect size

A standardized mean difference *Hedge’s g*, a modification of *Cohen’s d* (Vøllestad et al., 2012), was used as the effect size of the intervention. *The Hedge’s g* value was directly calculated using CMA 3.3 by inputting the sample sizes, post-test (or follow-up) means, and standard deviations of the intervention and control groups. Effect sizes were evaluated as followed: 0.2 for a small effect size, 0.5 for a medium effect size, and 0.8 for a large effect size (Kallapiran et al., 2015).

The random effects model was used for effect size calculation, and the mixed model was used for moderator variable analysis. The main theoretical rationale for using the random effects model are as follows: (1) the random effects model assumes that each independent effect size is based on the aggregation of multiple true effect sizes, implying a degree of variation between independent effect sizes, and the outcome data varies due to the fact that they come from multiple independent studies. (2) Analysis based on the random effects model allows for wider confidence intervals, reducing the risk of Type I error and can assign greater weight to small sample studies (Berkeljon and Baldwin, 2009). Meanwhile, for data analysis, *Q* and *I^2^* were used to evaluate heterogeneity, and *I^2^* refers to the proportion of the variance among studies in the overall variance (*I^2^* = 25, 50, 75%: low, medium, and high heterogeneity); when *Q* is significant and *I*^2^ ≥ 75%, it shows substantial heterogeneity among studies, justifying the use of a random effects model.

#### Publication bias assessment

The risk of publication bias was initially assessed using the funnel plot with the fail-safe number (Nfs) method, and further examined by Egger linear regression. If the funnel plot is a subjective evaluation of publication bias, and generally the data is distributed symmetrically around the center and the top, the likelihood of publication bias is low. However, the funnel plot assessment generally requires at least 10 included studies. If the intercept from Egger linear regression is close to 0 and insignificant, it suggests a low likelihood of publication bias (Egger et al., 1997); Nfs refers to the minimum value of the number of studies that make the existing conclusions “non-significant,” and the larger the Nfs, the lower the possibility of publication bias. When Nfs is less than 5 k + 10 (where k is the number of original studies), publication bias should be alerted (Rothstein et al., 2005).

#### Sensitivity analysis

Performed to assess result robustness, as variations in inclusion criteria, data extraction, or missing-data handling can influence meta-analytic outcomes (Borenstein et al., 2021). Outliers (studies with 95% CIs excluding the pooled effect size’s CI) were removed (Morgan et al., 2018).

#### Moderator analysis

To explore potential sources of heterogeneity, subgroup analysis and meta-regression analysis were conducted on anxiety and depression symptoms. The variables analyzed in subgroup analyses were disease type and scale type. Research showed that mental state varies depending on whether a disease is life-threatening, with significant differences in negative emotions among participants with different diseases (Chang and Chen, 2023; Curtis et al., 2004). Therefore, this study categorized diseases into non-life-threatening and life-threatening to investigate whether TCM Emotional Nursing has different effects on anxiety and depression caused by these two categories of diseases.

Apart from this, factors such as the type of scale used to assess the outcome variables (Li et al., 2023; Sun et al., 2023), sample size (Fox et al., 2020; Liu et al., 2021; White et al., 2019), year of study (Peng et al., 2023), participants’ age (Fuller and Kaiser, 2020), intervention duration, and disease course (Liu et al., 2021; Peng et al., 2023; Zhang et al., 2023) all had a significant effect on the intervention. Moderating variables on the effect of the intervention was also a primary objective of this study. Disease type and scale type are categorical variables and were explored through subgroup analysis, while sample size, year of study, age of participants, intervention duration, and course of disease were continuous variables and were analyzed by meta-regression analysis to assess their impact on intervention.

## Results

### Characteristics of studies included in the meta-analysis

The flow diagram of the study is shown in Figure 1. A total of 39 literatures with 40 independent trials met the inclusion criteria. Among which Wang et al. (2017) investigated the effects of one-week and two-week interventions, which were included as two separate studies. All included articles were in Chinese, with a total sample size of 4,425 participants. The patients had a diverse range of illnesses, including hypertension, coronary heart disease, bone fracture, and cancer. The patients aged from 60 to 75 years. The mental state of the patients was measured by scales with good reliability and validity, such as Self-Rating Anxiety Scale (SAS), Self-Rating Depression Scale (SDS), Hamilton Anxiety Scale (HAMA), Hamilton Depression Scale (HAMD). Detailed coding information is provided in Supplementary Table 1.

### Effect sizes for TCM emotional nursing on improving emotional state, anxiety, and depression

Table 1 shows effect sizes for TCM emotional nursing on improving emotional state, anxiety, and depression. The improvements in emotional state (*g* = 2.23, 95% CI [1.90, 2.58]), anxiety (*g* = 2.14, 95% CI [1.79, 2.49]), and depression (*g* = 2.18, 95% CI [1.83, 2.54]) all demonstrate large effect sizes. Funnel plots, fail-safe N combined with Egger’s linear regression were applied to assess potential publication bias. As seen in the funnel plot (Supplementary Figure 1), the studies related to emotional state, anxiety, and depression were symmetrically distributed around the center, indicating a low risk of bias. The fail-safe N also suggested no risk of bias. However, the significant intercept in Egger’s linear regression indicated a potential risk of publication bias. Further trim-and-fill analysis found that the adjusted effect sizes for emotional state (*g* = 2.00, 95%CI [1.64, 2.36]) and depression (*g* = 2.18, 95%CI [1.83, 2.54]) showed no significant deviation from unadjusted estimates, indicating minimal impact of publication bias on conclusions.

**Table 1:** Effect size and publication bias test of TCM emotional nursing on improving the psychological state of elderly patients with psychosomatic disorders.

Outcome variables	*k*	*n*	Heterogeneity		Effect size		Publication bias			
							*N*_fs_	Egger’s regression test		
			*Q*_W_	*I* ^2^	*Hedge’g*	95% CI		Intercept	*t*	*p*
Emotional state	40	4,425	800.33^***^	95.13	2.23^***^	1.90,2.58	8,194	6.47	2.662	0.011
Anxiety	36	4,009	706.52^***^	95.05	2.14^***^	1.79,2.49	2,160	5.11	1.898	0.066
Depression	37	4,172	799.52^***^	95.50	2.18^***^	1.83,2.54	3,279	6.66	2.606	0.013

*k* represents the number of independent effect sizes, *n* is the sample size, and the 95% CI is the 95% confidence interval for the effect size g corresponding to the outcome variable; *p < 0.05, **p < 0.01, ***p < 0.001; heterogeneity test: *Q*^W^ represents the test statistic for within-group heterogeneity, two-tailed test.

Results of sensitivity analysis. In the test of emotional state as the outcome variable, one outlier was removed (Zhang et al., 2014), and the effect size was *g* = 2.28, 95%CI [1.94, 2.62]. For anxiety, one outliers were removed (Zhang et al., 2014), and the effect size was *g* = 2.19, 95%CI [1.84, 2.54]. For depression, one outliers were removed (Chen, 2013), and the effect size was *g* = 2.18, 95%CI [1.83, 2.54].

### Subgroup analysis of TCM emotional nursing to improve anxiety and depression in elderly patients

Subgroup analyses were conducted for anxiety and depression based on disease type and scale type, as shown in Tables 2, 3. In the subgroup analysis by diseases, whether the disease was life-threatening did not significantly affect the effects of nursing on anxiety and depression. In the subgroup analysis by scale type, the type of scale had a significant effect on the intervention effect for anxiety but not for depression. Specifically, the intervention effect assessed using the Self-Rating Anxiety Scale (SAS) was superior to that assessed using the Hamilton Anxiety Scale (HAMA) (*g* = 2.21 vs. *g* = 1.09, *p* < 0.001).

**Table 2:** Results of subgroup analysis of anxiety.

Subgroup	*k*	*Z*	*Q*_w_	*I* ^2^	*g*	95% CI	*p*
Disease type							0.603
Non life-threatening	9	24.277^***^	283.17^***^	97.16	1.95^***^	1.07,2.82	
Life-threatening	27	41.697^***^	421.23^***^	93.83	2.20^***^	1.83,1.57	
Scale type							<0.001
HAMA	1	5.62^***^	0	0	1.09^***^	0.71,1.28	
SAS	34	11.97^***^	670.06^***^	95.08	2.21^***^	1.85,2.58	

*k* represents the number of independent effect sizes, *n* is the sample size, and 95% CI is the 95% confidence interval for the effect size *g* corresponding to the outcome variable; *p < 0.05, ** *p* < 0.01, ***p < 0.001; heterogeneity test: QW stands for the test statistic for within-group heterogeneity, two-tailed test.

HAMA, Hamilton Anxiety Scale; SAS, Self-Rating Anxiety Scale.

**Table 3:** Results of subgroup analysis of depression.

Subgroup	*k*	*Z*	*Q*_w_	*I* ^2^	*g*	95% CI	*P*
Disease type							0.804
Non life-threatening	10	5.079^***^	297.39^***^	96.97	2.10^***^	1.288,2.906	
Life-threatening	27	10.858^***^	502.12^***^	94.82	2.21^***^	1.812,2.610	
Scale type							0.404
HAMA	3	3.656^***^	27.84^***^	92.82	1.82	0.843,2.792	
SAS	33	11.340^***^	752.62^***^	95.75	2.26	1.873,2.656	

*k* represents the number of independent effect sizes, *n* is the sample size, and 95% CI is the 95% confidence interval for the effect size g corresponding to the outcome variable; *p < 0.05, **p < 0.01, ***p < 0.001; heterogeneity test: QW stands for the test statistic for within-group heterogeneity, two-tailed test.

HAMA, Hamilton Anxiety Scale; SAS, Self-Rating Anxiety Scale.

### Meta-regression of TCM emotional nursing to improve anxiety and depression in elderly patients

The effects of sample size, year of study, age of participants, and disease duration on the effect size of anxiety and depression care are shown in Tables 4, 5. In the meta-regression model for anxiety, the *R*^2^ value was 0.33, indicating that these variables explained 33% of the variance in effect sizes. Sample size had a significant effect on effect size (*p* = 0.0394) and age had a marginally significant effect (*p* = 0.0603). In the meta-regression model for depression, the *R*^2^ was 0.98, showing that the included moderator variables explained 98% of the variance in effect size. Sample size (*p* < 0.001), age (*p* = 0.0003), and disease duration (*p* = 0.0023) all had significant effects on effect size. It’s worth noting that the effect of intervention period on the effect size was not analyzed due to the insufficient data on intervention period.

**Table 4:** Meta-regression for anxiety.

Variables	*b*	95% CI	SE	*z*	*p*
Sample size	0.0055	0.0003, 0.0106	0.0026	2.06	0.0394
Study year	−0.059	−0.263, 0.1450	0.1041	−0.57	0.5709
Age	−0.1787	−0.3651, 0.0077	0.0951	−1.88	0.0603
Duration of disease	0.0776	−0.1444, 0.2996	0.1133	0.69	0.4933
*R* ^2^	0.33				
*Q* (df)	6.66^***^ (4)				

^***^p < 0.001.

**Table 5:** Meta-regression for depression.

Variables	*b*	95% CI	SE	*z*	*p*
Sample size	0.0054	0.0039, 0.0068	0.0008	7.12	<0.001
Study year	−0.0406	−0.1059, 0.0247	0.0333	−1.22	0.2234
Age	−0.1124	−0.1733, −0.0516	0.0311	−3.62	0.0003
Duration of disease	0.1125	0.0403, 0.1847	0.0368	3.05	0.0023
*R* ^2^	0.96				
*Q* (*df*)	53.18^***^ (4)				

^***^p < 0.001.

### Study quality assessment

**Figure 2:**
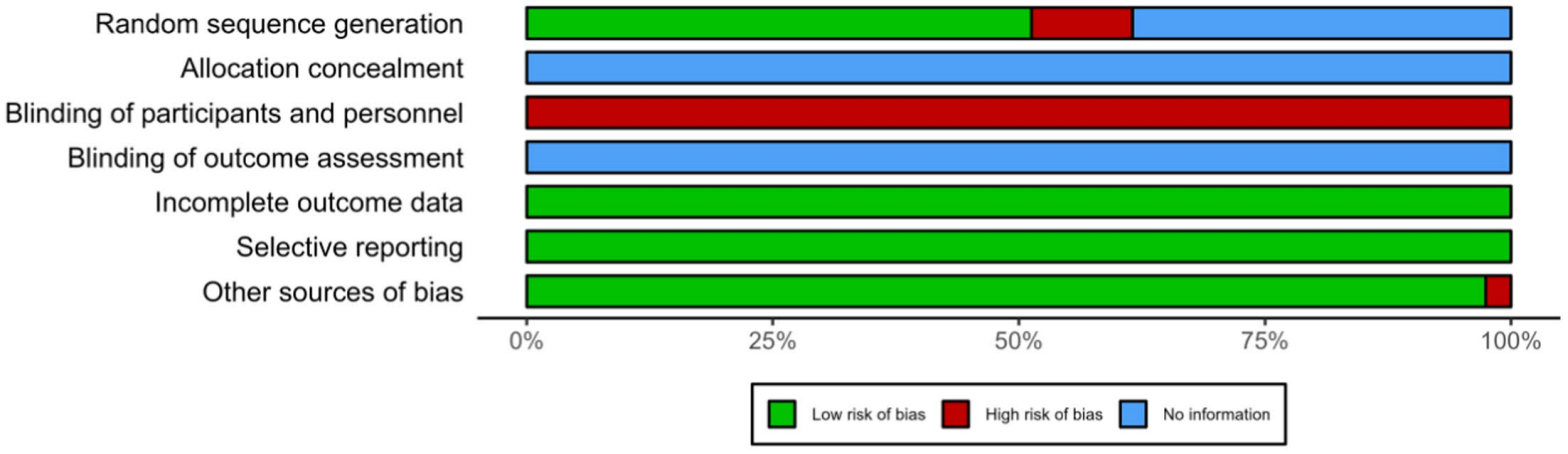
Quality assessment of included study.

Figure 2 provides a summary of the risk of bias for the included studies. The risk of bias assessment for each study specifically can be found in Supplementary Figure 2.

As illustrated, most of the studies had adequate random sequence generation, but did not specify how allocation concealment was performed, leading to an unclear risk in allocation concealment. It is evident that more clearly that there is a high risk of blinding during implementation, as well as an unknown risk. The data and choices reported on the uncompleted intervention were low risk.

Overall, no study was found to have a high risk of bias on more than three domains, so the risk level included in this meta-analysis is considered moderate.

## Discussion

Set against the backdrop of an aging population in China, this study investigated the effects of TCM emotional nursing interventions on depression and anxiety in elderly patients. Through subgroup analysis and meta-regression analysis, associating factors of the intervention effects were also explored. The findings provided empirical evidence to support the tailored clinical application of TCM emotional nursing, facilitating more effective interventions.

The results of this study indicate that TCM emotional nursing significantly reduces anxiety and depression in elderly patients with physical and mental illnesses, with all effects demonstrating large effect sizes. Previous studies on TCM emotional nursing have shown its effectiveness in improving depressive disorders (Liu et al., 2021), postoperative emotions in breast cancer patients (Xiao and Peng, 2023), anxiety symptoms in cancer patients (Kang et al., 2017), and anxiety and depression in stroke patients (Li et al., 2020; Liang et al., 2015; Niu, 2020). However, the intervention effect size obtained in this study is significantly larger than those reported in other similar studies. Among comparable studies with measurable effect sizes, a meta-analysis exploring the effects of TCM emotional nursing on improving depression in stroke patients indicated an intervention effect smaller than the large effect size observed in this study, and the subjects of that study were not limited to older adults (Niu, 2020). Some non-traditional cultural intervention methods, such as Western theory-based psychological counseling, exercise interventions, and physical activity, demonstrated small to moderate effect sizes in alleviating depression and anxiety among older adults (SMD = −0.39 ~ 0.60; Goodarzi et al., 2024; Orgeta et al., 2017; Soong et al., 2025). In contrast, interventions rooted in traditional culture exhibited larger effect sizes, such as Tai Chi intervention (SMD: −1.19 for anxiety; −0.65 for depression, Kuang et al., 2024) and Baduanjin intervention (Hedge’s *g* = −0.99 for anxiety; −1.07 for depression, Zou et al., 2018). This suggests that the substantial intervention effect observed in this study may be attributed to older adults’ cultural identification with traditional Chinese practices, as the trust and expectations fostered by such identification are also recognized as therapeutic factors in psychotherapy (Wampold, 2015). Additionally, many TCM interventions are administered over extended periods (weeks to months), allowing for cumulative physiological and psychological adjustments.

This study explored the effects of participants’ age, type of scale used, disease type (life-threatening vs. non-life-threatening), sample size, year of study, and disease duration on the effectiveness of care. The results of the meta-regression analysis indicated that age had a significant negative impact on intervention outcomes. This further demonstrates that psychological characteristics of patients vary across different age groups, leading to differing responses to interventions (Fuller and Kaiser, 2020). Younger elderly patients exhibited better outcomes from TCM emotional nursing, possibly due to their higher acceptance of the therapy, which allows them to benefit more from emotional care interventions. Therefore, targeted nursing should be implemented for patients of different ages to ensure optimal outcomes (Ma, 2019). The study found that the duration of the disease had a significant effect on the intervention’s impact on depression, with longer duration correlating with better outcomes. This finding contradicts previous research, which generally suggests that longer duration of psychological illness are associated with poorer prognoses (Hu et al., 2020). The possible reason for this result is that patients with longer duration of disease may have adapted to the physical conditions, and the associated psychological impacts (Kübler-Ross, 2004). Increased understanding of their physiological illnesses could improve their psychological state, which, when coupled with interventions, may further enhance mental health.

In addition to the above factors affecting the effectiveness of care, study-related factors such as sample size and type of scale used also influenced the effectiveness of nursing. Sample size showed a positive correlation with intervention outcomes, aligning with similar studies indicating that sample size can lead to result bias (Liu et al., 2021). Regarding the improvement of anxiety in elderly patients, the effect measured by SAS demonstrated better outcomes compared to that measured by the HAMA, which is likely due to the stricter criteria for positive detection inherent to the scales and highlights the limitations of self-report measures.

Although this study drew some conclusions that can guide the clinical practice of TCM emotional nursing, there are several limitations:

(1) The quality of the included studies was limited. For instance, none of the studies elaborated on allocation concealment or blinding in outcome assessment. Additionally, blinding of implementers and patients during the intervention was not performed. However, it is acknowledged that double-blinding of participants and researchers is difficult to achieve in clinical interventions (Benz et al., 2020). (2) The included studies exhibited high heterogeneity and potential publication bias. Although the included studies employed TCM emotional nursing, variations in the specific implementation of interventions and differences in intervention protocols across studies may have contributed to the high heterogeneity. Future research could use component meta-analysis to code different intervention components and explore their effects. Bias assessment indicated potential publication bias, likely due to the predominance of positive results in the included studies (Liang et al., 2015). (3) Data miss from the included studies might reduce the reliability of the findings. Most of the included studies did not clearly report the intervention duration, which led to difficulties in data extraction and analysis, and directly affecting the reliability of the results.

In conclusion, the results of this study are affected by the quality of the study to a certain extent, but the overall findings demonstrate that TCM emotional nursing exerts a positive effect on the psychological state of elderly patients. Regardless of whether the disease is life-threatening, TCM emotional nursing consistently plays a stabilizing role. Therefore, it is worth widespread application in the clinical care of elderly patients.

## Data Availability

The original contributions presented in the study are included in the article/Supplementary material, further inquiries can be directed to the corresponding author.
