# Supplementary material for: A meta-analysis on the effectiveness of Traditional Chinese Medicine Emotional Nursing in improving anxiety and depression symptoms in elderly patients
Source: Front Psychol. 2025 Jun 19;16:1535349. doi: 10.3389/fpsyg.2025.1535349 (PMC12222280; doi:10.3389/fpsyg.2025.1535349)
Supplement: Supplementary file 1 [file Supplementary_file_1.docx]

**A meta-analysis on the effectiveness of Traditional Chinese Medicine Emotional Nursing in improving anxiety and depression symptoms in elderly patients Supplementary Material**

Content

[Table 1 Characteristics of studies included in the meta-analysis 1](#_Toc29762)

[Figure 1 Bias risk assessment funnel plot 5](#_Toc19310)

[Figure 2 Quality assessment for each study 7](#_Toc27149)

[Included study 7](#_Toc13370)

# Table 1 Characteristics of studies included in the meta-analysis

| No. | | Autor(Year) | Research year | Sample size(experimental group,control group) | Disease style | Age of participants | Disease course | Result variables | Measurement tool |
| --- | --- | --- | --- | --- | --- | --- | --- | --- | --- |
| 1 | Na Xie(2023)^[1]^ | | June,2020-June,2021 | 120（60，60） | Coronary heart disease | 71.70 | 1-11years | Anxiety、Depression | HAMA、HAMD |
| 2 | Youyu Cheng(2022)^[2]^ | | August,2019-January,2020 | 98（49，49） | Hypertension | 66.97 | / | Anxiety | SAS |
| 3 | Ling Yang(2022)^[3]^ | | 2021-2022 | 90（45，45） | Hearing impaired | 70.23 | / | Emotional state | SAS、SDS |
| 4 | Shanshan Sun et al (2022)^[4]^ | | November,2020-November,2021 | 200（100，100） | Cerebral arterial thrombosis | 67.40 | / | Anxiety、Depression | SAS、SDS |
| 5 | Chunxue Ren(2021)^[5]^ | | February,2019-June,2020 | 74（34，34） | Pulmonary heart disease | 74.22 | / | Anxiety、Depression | SAS、SDS |
| 6 | Jixia Cheng(2021)^[6]^ | | November,2018-November,2020 | 98（49，49） | Fracture | 71.6 | / | Anxiety、Depression | SAS、SDS |
| 7 | Rongrong Ma et al(2021)^[7]^ | | February,2019-January,2020 | 100（50，50） | Stable angina pectoris | 74.84 | 5-8years | Anxiety、Depression | SAS、SDS |
| 8 | Xiaoying Zhang(2021)^[8]^ | | September,2018-September,2020 | 80（40，40） | Hypertension | 70.7 | 14-88months | Anxiety、Depression | SAS、SDS |
| 9 | Yue Wang(2021)^[9]^ | | May,2018-March,2019 | 86（44，42） | Chronic obstructive pulmonary disease | 69.25 | 1-9years | Anxiety、Depression | HAMA、HAMD |
| 10 | Yuehua Chang(2021)^[10]^ | | 19^th^,November,2017-19^th^,November,2020 | 130（65，65） | Pulmonary heart disease | 73.55 | / | Anxiety、Depression | SAS、SDS |
| 11 | Xiujuan Jiang et al(2021)^[11]^ | | August,2018-September,2020 | 136（68，68） | Fracture | 64.99 | / | Anxiety、Depression | SAS、SDS |
| 12 | Juanjuan Yang et al(2020)^[12]^ | | October,2019-October,2020 | 80（40，40） | Malignant tumor | 64.9 | / | Anxiety、Depression | SAS、SDS |
| 13 | Liqiong Lv et al(2020)^[13]^ | | January,2018-September,2019 | 112（56，56） | Lung cancer | 64.95 | / | Anxiety、Depression | SAS、SDS |
| 14 | Xiuqin Hong et al(2020)^[14]^ | | February,2017-October,2018 | 86（43，43） | High biood pressure |  | 2-22years | Anxiety、Depression | SAS、SDS |
| 15 | Shaoli Jia(2020)^[15]^ | | May,2018-July,2019 | 60（30，30） | Cerebral apoplexy | 66.7 | / | Anxiety、Depression | SAS、SDS |
| 16 | Xiaoyan Li(2020)^[16]^ | | March,2016-March,2019 | 68（34，34） | Diabetes with depression | 71.7 | / | Anxiety、Depression | HADS |
| 17 | Minong Zhang et al(2020)^[17]^ | | July,2018-December,2018 | 80（40，40） | Patients in ICU | 67.16 | / | Anxiety、Depression | SAS、SDS |
| 18 | Shan Zhou(2020)^[18]^ | | January,2019-January,2020 | 90（45，45） | Coronary heart disease | 69.78 | 3-4years | Anxiety、Depression | SAS、SDS |
| 19 | Yuxia Zhu(2020)^[19]^ | | March,2018-March,2019 | 73（37，36） | Fracture of neck of femur | 71.51 | / | Anxiety、Depression | SAS、SDS |
| 20 | Jiangyu Xie(2020)^[20]^ | | October,2019-December,2019 | 72（36，36） | Insomnia | 69.38 | 5-7years | Anxiety、Depression | SAS、SDS |
| 21 | Meizhu Hu(20190^[21]^ | | May,2016-June,2017 | 88（44，44） | Hypertension | 64.5 | / | Anxiety、Depression | SAS、SDS |
| 22 | Jialing Yan(2019)^[22]^ | | February,2017-February,2019 | 90（45，45） | Hypertension | 71.73 | 3-12years | Depression | SDS |
| 23 | Yan Chen(2019)^[23]^ | | December,2017-December,2018 | 150（75，75） | Hypertension | 73.00 | / | Anxiety、Depression | SAS、SDS |
| 24 | Ru Chen(20180^[24]^ | | July,2016-July,2017 | 100（50，50） | Pulmonary heart disease | 69.35 | 6months-10years | Anxiety、Depression | SAS、SDS |
| 25 | Rong Zuo(2018)^[25]^ | | September,2016-September,2017 | 116（58，58） | Diabetic patients with depression | 64.7 | / | Depression | HAMD、SDS |
| 26 | Songru Liu(2018)^[26]^ | | October,2016-October,2017 | 120（60，60） | Hypertension | 74.93 | / | Anxiety、Depression | SAS、SDS |
| 27 | Ning Pei(2018)^[27]^ | | October,2011-October,2017 | 500（250，250） | Coronary heart disease | 76.45 | 8-10years | Anxiety、Depression | SAS、SDS |
| 28 | Wuyan Sun(2017)^[28]^ | | January,2017-November,2017 | 90（45，45） | Hypertension | 68.07 | 12-15years | Anxiety、Depression | SAS、SDS |
| 29 | Lei Wang et al(2017)^[29]^ | | June,2013-June,2015 | 86（43，43） | Primary liver cancer | 66.04 | / | Anxiety、Depression | SAS、SDS |
| 30 | Zhiying Yang et al(2017)^[30]^ | | January,2016-March,2017 | 90（45，45） | Hypertension | 68.89 | 1-15years | Anxiety、Depression | SAS、SDS |
| 31 | Hua Ji(2016)^[31]^ | | July,2014-December,2016 | 126（63，63） | Hypertension | 76.52 | 3-19years | Anxiety、Depression | SAS、SDS |
| 32 | Junpeng Chen(2016)^[32]^ | | April,2013-April,2015 | 96（48，48） | Hypertension | 78.65 | / | Anxiety | SAS |
| 33 | Shuling Li(2016)^[33]^ | | July,2014-March,2016 | 74（37，37） | Hypertension | 69.43 | / | Anxiety、Depression | SAS、SDS |
| 34 | Guofei Wang et al(2015)^[34]^ | | / | 100（50，50） | Hypertension | 67.8 | 3-15years | Anxiety、Depression | HAMA、HAMD |
| 35 | Yujuan Cheng et al(2015)^[35]^ | | September,2010-September,2014 | 327（164，163） | Senile pruritus | / | / | Anxiety、Depression | SAS、SDS |
| 36 | Qiongzhong Li et al(2014)^[36]^ | | January,2011-October,2013 | 100（50，50） | Hip fracture | 71.6 | / | Anxiety、Depression | SAS、SDS |
| 37 | Qian Zhang et al(2014)^[37]^ | | October,2013-March,2014 | 55（27，28） | Senile pruritus | 70.53 | 4-6months | Anxiety、Depression、Negative emotion | SAS、SDS、GDS |
| 38 | Xia Chen(2013)^[38]^ | | June,2011-June,2012 | 120（60，60） | Pulmonary heart disease | 69.82 | 5-8years | Depression | SDS |
| 39 | Xuehua Chen et al(2010)^[39]^ | | March,2007-March,2010 | 60（30，30） | Hypertension | 79 | / | Anxiety、Depression | SAS、SDS |

Note：The control group was treated with routine nursing；Only the study of Rongrong Ma et al.(2021)specifically mentioned that the intervention period was 3 months ,and the other studies did not specify the intervention period.

SAS：Self-Rating Anxiety Scale、SDS：Self-Rating Depression Scale、HAMA：Hamilton Anxiety Scale、HAMD：Hamilton Depression Scale、HADS：Hospital Anxiety and Depression Scale、GDS：The Geriatric Depression Scale。/：This content is not mentioned in the studies.。

Funnel plot of Mood state (k=40,n=4425)

Funnel plot of anxiety(k=36,n=4009)

Funnel plot of depression(k=37,n=4172)

Figure 1 Bias risk assessment funnel plot


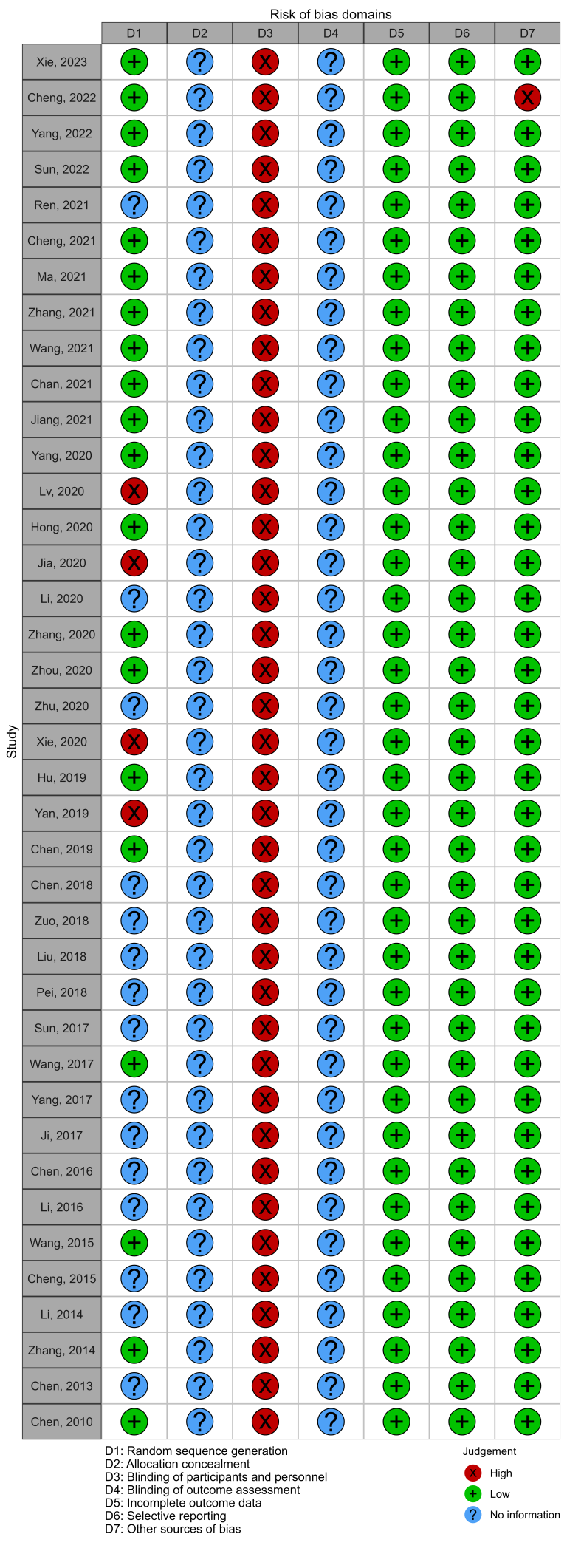


# Figure 2 Quality assessment for each study

# Included study

[1] Xie N.Influence of Traditional Chinese Emotional Nursing on the Negative Emotions of Elderly Patients with Coronary Heart Disease and Angina Pectoris[J].*Yi Shou Bao Dian*,2023(2):131-133

[2] Cheng YY.Effect of Remote Emotional Nursing of Traditional Chinese Medicine on Elderly People with Normal Hypertension or Hypertension[J].*Journal of Jiangxi University of CM*,2022,34(4):46-49

[3] Yang L.Application Status and Thinking of TCM Emotional Nursing in Elderly Hearing Impairment Health Care Population[J].*Chinese Medicine Digest:Otorhinolaryngology*,2022,37(4):173-174, 177

[4] Sun SS,Sun F,Liu L,et al.Influence of Traditional Chinese Medicine Emotional Rehabilitation Nursing on Emotion of Elderly Patients with Cerebral Arterial Thrombosis[J].Journal of *Qilu Nursing*,2022,28(15):152-154

[5] Ren CX.Emotional Nursing Effect of Traditional Chinese Medicine in Nursing of Elderly Patients[J].*Guide of China Medicine*,2021,19(6):186-187

[6] Cheng JX.Influence of Traditional Chinese Emotional Nursing on Negative Emotions and Sleep Quality of Elderly Orthopedic Patients after Surgery[J].*Chinese Remedies&Clinics* ,2021,21(8):1434-1436

[7] Ma RR,Zhi Hong Liu.Influence of traditional Chinese emotional nursing on bad mood and quality of life in elderly patients with angina pectoris[J].*Guangming Journal of Chinese Medicine*,2021,36(8):1338-1340

[8] Zhang XY.Application of TCM Emotional Nursing in Elderly Patients with Hypertension[J].*Guangming Journal of Chinese Medicine*,2021,36(19):3358-3360

[9] Wang Y.Application Effect of TCM Emotional Nursing in Elderly Patients with Chronic Obstructive Pulmonary Disease[J].*Medical Journal of Chinese People`s Health*,2021,33(10):172-174

[10] Chang YH.Effect of Traditional Chinese Medicine Emotional Nursing in Elderly Patients with Pulmonary Heart Disease[J].Chinese Science and Technology Periodical Database (full version) Pharmaceutical and Health Care, 2021(1):160-161

[11] Jiang XJ,Qi CP,Ju CB,et al.Influence of Traditional Chinese Emotional Nursing on Negative Emotions and Sleep Quality in Elderly Orthopedic Patients after Surgery[J].*Chinese Health Care*,2021,39(13):89-91

[12] Yang JJ,Liu Y,Leng AH,et al.Effect of TCM Emotion Differentiation Nursing on Emotion,Sleep and Health Behavior of Middle-aged and Elderly Patients with Malignant Tumor[J].*World Journal of Sleep Medicine*,2020,7(12):2076-2078

[13] Lv LQ,Ma YQ,Tang X,et al.The Influence of Traditional Chinese Medicine Emotional Nursing on the Negative Emotion and Quality of Life of Elderly Patients with Lung Cancer[J].*Chinese and Foreign Medicine Research* ,2020,18(8):99-101

[14] Hong XQ,Shu Zhen Su.Influence of Traditional Chinese Emotional Nursing on Bad Mood and Health Belief of Elderly Patients with Hypertension[J].Prevention and Treatment of Cardiovascular Disease,2020,10(7):69-71

[15] Jia SL.Influence of Emotional Nursing of Traditional Chinese Medicine on Emotion and Rehabilitation Compliance of Elderly Stroke Patients[J].*Electronic Journal of Clinical Medical Literature*,2020,7(31):92

[16] Li XY.Application Effect of TCM Emotional Nursing on Elderly Diabetic Patients with Depression[J].*Tang Niao Bing Xin Shi Jie*,2020,23(13):158-160

[17] Zhang MN,Yin AS,Yang ZT,et al.Observation on the Effect of Traditional Chinese Medicine Emotional Nursing in the Elderly Patients in ICU[J].*Chinese Community Doctors*,2020,36(02):148-150

[18] Zhou S.Influence of Traditional Chinese Emotional Nursing on Negative Emotions and Quality of Life in Elderly Patients with Coronary Heart Disease[J].*Healthmust-Readmagazine*,2020(33):189

[19] Zhu YX,Xie XJ,Xu XP,et al.Influence of Emotional Nursing in Traditional Chinese Medicine on Negative Emotions in Elderly Patients with Hip Fracture[J].*Yi Yao Qian Yan*,2020,10(31):201-202

[20] Xie JY.Influence of Traditional Chinese Emotional Nursing on Sleep Treatment and Psychological Status of Elderly Insomniacs[J].*Healthmust-Readmagazine*,2020(20):171

[21] Hu MZ.Observation on the Effect of TCM Emotional Nursing in Elderly Patients with Hypertension[J].*Journal of Clinic Nursing`s Practicality*,2019,4(03):53-54

[22] Yan JL.Study on the Influence of Traditional Chinese Emotional Nursing on the Quality of Life of Elderly Patients with Hypertension[J].*Health Care Guide*,2019(35):279

[23] Chen Y.Study on the Application of Traditional Chinese Emotional Nursing in Improving Bad Mood and Health Belief in Elderly Patients with Hypertension[J].*Healthmust-Readmagazine*,2019(1):5

[24] Chen R,Zhao LL,Lv LN,et al.A Study on the Effect of TCM Emotional Nursing on Senile Patients with Pulmonary Heart Disease[J].*Clinical Journal of Chinese Medicine*,2018,10(03):113-115

[25] Zuo R.Application Effect of Emotion Nursing of Traditional Medicine on Elderly Diabetic Patients with Depression [J].*Chinese Community Doctor*,2018,34(18):142-143

[26] Liu SR.Application Effect of Traditional Chinese Emotional Nursing in Improving Bad Mood and Health Belief in Elderly Patients with Hypertension[J].*Guangming Journal of Chinese Medicine*,2018,33(24):3739-3741

[27] Pei N.Application Value of TCM Emotional Nursing in Elderly Patients with Coronary Heart Disease[J].*Healthmust-Readmagazine*,2018(22):140-141

[28] Sun WY.Study on the Effect of Traditional Chinese Medicine Emotional Nursing on Elderly Patients with Hypertension[J].*Contemporary Medical Symposium*,2017,15(23):211-213

[29] Wang L,Chen CX,Xia CL,et al.Prospective Controlled Study on TCM Emotional Nursing on the Bad Mood and Survival Period of Elderly Patients with Liver Cancer[J].*Chinese General Practice Nursing* ,2017,15(09):1067-1069

[30] Yang ZY,Xie J,Chen Y,et al.Influence of Emotional Nursing of Traditional Chinese Medicine on Blood Pressure and Psychological State in Elderly Patients with Hypertension[J]*.Journal of Clinic Nursing`s Practicality*,2017,2(50):58-60

[31] Ji H.Study on the Application of TCM Emotional Nursing on Elderly Patients with Hypertension[J].*Health Care Guide*,2017(27):152

[32] Chen JP.Effect Analysis of Traditional Chinese Medicine Emotional Nursing on Elderly Patients with Hypertension[J].*Guide of Chinese Medicine*,2016,14(01):210

[33] Li SL.Clinical Study on Emotional Nursing of Traditional Chinese Medicine in Elderly Patients with Hypertension[J].*Nei Mongol* *Journal of Traditional Chinese*,2016,35(13):172-173

[34] Wang GF,Ying Jun Xu.Explore the Application of Traditional Chinese Medicine Emotional Nursing in Elderly Patients with Hypertension[J].*Liaoning Journal of Traditional Chinese Medicine*,2015,42(05):1102-1104

[35] Cheng YJ,Hou YF,Zi SX,et al.Application Effect of TCM Emotional Nursing in Elderly Patients with Pruritus[J].*Chinese Journal of Goal Industry Medicine*,2015,18(09):1585-1587

[36] Li QZ,Zeng GT,Chen CL,et al.Observation on the Effect of Traditional Chinese Medicine Emotional Nursing on the Psychological Characteristics of Elderly Hip Fracture[J].*Journal of Qiqihar University of Medicine*,2014,35(11):1689-1690

[37] Zhang Q,Gao J,Shi LH,et al.Study on the Effect of Traditional Chinese Medicine Emotional Nursing on Improving the Negative Emotions of Senile Patients with Pruritus[J].*Chinese Journal of Convalescent Medicine*,2014,23(12):1066-1068

[38] Chen X.Experience of Traditional Chinese Emotional Nursing in Patients with Senile Pulmonary Heart Disease[J].*Journal of New Chinese Medicine*,2013,45(06):214-215

[39] Chen XH,Zheng LJ,Chen FX,et al.Observation on Therapeutic Effect of Traditional Chinese Medicine Emotional Nursing Intervention in Elderly Patients with Hypertension[J].*Fujian Journal of TCM*,2010,41(05):60-61
